# Examining the Relationship Between Rheumatoid Arthritis, Multimorbidity, and Adverse Health‐Related Outcomes: A Systematic Review

**DOI:** 10.1002/acr.24587

**Published:** 2022-05-27

**Authors:** Jordan Canning, Stefan Siebert, Bhautesh D. Jani, Louisa Harding‐Edgar, Isla Kempe, Frances S. Mair, Barbara I. Nicholl

**Affiliations:** ^1^ University of Glasgow Glasgow UK

## Abstract

**Objective:**

Multimorbidity (the coexistence of two or more long‐term conditions) is highly prevalent in people who have rheumatoid arthritis (RA). The present work systematically reviewed the literature to determine the effect of multimorbidity on all‐cause mortality, functional status, and quality of life in RA.

**Methods:**

Six electronic databases were searched: CINAHL, The Cochrane Library, Embase, Medline, PsycINFO, and Scopus. Full‐text longitudinal observational studies in English were selected. Quality appraisal of studies was undertaken using the Cochrane‐developed QUIPS tool and a narrative synthesis of findings conducted.

**Results:**

The search strategy identified 5,343 articles, with 19 studies meeting the inclusion criteria. Nine studies had mortality as an outcome, 9 reported functional status and/or quality of life, and 1 study reported both mortality and functional status. The number of participants ranged from 183 to 18,485, with studies conducted between 1985 and 2018. The mean age of participants ranged from 52.0 to 66.6 years, and 60.0–88.0% were female. Nine studies showed a significant association between multimorbidity and higher risk of mortality in people with RA. Ten studies reported significant associations between multimorbidity and reduced functional status in RA. Three studies also showed a further association with reduced quality of life. Only one study investigated the influence of mental health comorbidities on outcomes.

**Conclusion:**

Our review findings indicate that multimorbidity is a significant predictor for higher mortality and poorer functional status/quality of life in people with RA and should be considered in clinical management plans.

## INTRODUCTION

Rheumatoid arthritis (RA) is the most common autoimmune inflammatory arthropathy, with an estimated global prevalence of ~20 million people (1). RA is characterized by pain, swelling, and stiffness of the synovial joints that can significantly impair physical functioning, activities of daily living, and overall quality of life (2). Improved therapies and treatment guidelines, including the development of biologic disease‐modifying antirheumatic drugs and the implementation of treat‐to‐target initiatives, have greatly improved clinical outcomes for many individuals with RA (3). However, extraarticular manifestations of RA and the presence of other long‐term conditions (LTCs) can cause further complications.

**SIGNIFICANCE & INNOVATIONS**

- To date, most research in this area has focused on rheumatoid arthritis (RA) and one other long‐term condition (comorbidity) rather than RA and ≥2 conditions (multimorbidity).
- We are the first, to our knowledge, to systematically review the existing literature in order to establish the association between RA, multimorbidity, and the following health‐related outcomes: all‐cause mortality, functional status, and quality of life.
- We show that multimorbidity is significantly associated with higher mortality, poorer functional status, and lower quality of life in people who have RA.
- Health care professionals should pay close attention to multimorbidity when treating people who have RA, and further research on the impact of concurrent mental health conditions in RA is needed.

Comorbidity (the existence of any additional LTC together with an index condition [4]) is well‐documented in RA, with the average person with RA having 1.6 additional LTCs (5). Common comorbid conditions in RA include cardiovascular disease (CVD), hypertension, osteoporosis, and depression (6), which are strongly associated with higher health care utilization, premature mortality, and poorer health‐related outcomes (7). Likewise, multimorbidity (the coexistence ≥2 LTCs [8]) itself is associated with adverse outcomes. However, less is known about the impact of multimorbidity in people with RA despite rheumatic and musculoskeletal diseases being a major presence in multimorbidity in the general population (9). The present systematic review explored the literature to determine the effect, if any, of multimorbidity on mortality and other health‐related outcomes (specifically functional status and quality of life) in people who have RA.

## MATERIALS AND METHODS

### Protocol and registration

We have previously published a detailed systematic review protocol (10), which followed the Preferred Reporting Items for Systematic Reviews and Meta‐Analyses (PRISMA) guidelines (11). The review is also registered on the International Prospective Register of Systematic Reviews (PROSPERO registration no. CRD42019137756).

### Eligibility criteria

We aimed to identify longitudinal observational studies with extractable quantitative data relating to multimorbidity (exposure) and all‐cause mortality, functional status, or quality of life (outcomes) in adults with RA (population) with no comparator(s). Studies were full‐text articles published in the English language with no restriction on publication date. Full inclusion and exclusion criteria are summarized in Supplementary Table 1, available on the *Arthritis Care & Research* website at http://onlinelibrary.wiley.com/doi/10.1002/acr.24587.

### Information sources

Studies were sourced by searching electronic medical databases using a comprehensive search strategy. This was applied to the Cumulative Index to Nursing & Allied Health Literature (CINAHL; EBSCOhost; 1982–present), Cochrane Library (Central Register of Controlled Trials [CENTRAL]); Wiley; 1996–present), Embase (Ovid; 1947–present), Medline (Ovid; 1946–present), PsycINFO (EBSCOhost; 1806–present), and Scopus (Elsevier; 1996–present). The search included studies published until December 2, 2020.

### Search

The search strategy combined 4 key themes: RA (1), comorbidity/multimorbidity (2), mortality (3), and functional status/quality of life (4), using predefined search terms (10).

### Study selection

Duplicate citations obtained from the database search were removed, and all remaining citations were uploaded to the systematic review management software, DistillerSR (Evidence Partners; Ontario, Canada). The titles and abstracts of these studies were screened against predefined criteria (10), followed by full‐text screening of eligible studies. This process was performed by the primary researcher (JC) and one other member of the review team (SS, BDJ, LH‐E, IK, FSM, or BIN) in an independent manner. Disagreements were resolved through discussion and consultation with a third reviewer when necessary. A citation/reference list search of included studies was also performed to identify any additional relevant studies but yielded no further eligible texts.

### Data extraction process

The data extraction process was performed in duplicate by the primary researcher (JC) and another member of the review team (SS, BDJ, LH‐E, IK, FSM, or BIN) using a predefined data extraction form (10). Discrepancies in data were again resolved through discussion and/or consultation with a third reviewer.

### Data items

The data extraction form followed a prespecified framework covering population, exposure, comparator, and outcomes (see above for eligibility criteria), based on Cochrane Handbook guidance (12). Details related to these data items were extracted in addition to study characteristics.

### Quality appraisal of included studies

The risk of bias in individual studies was assessed independently by 2 reviewers (JC and SS, BDJ, LH‐E, IK, FSM, or BIN) using the Cochrane Methods Group–developed Quality in Prognostic Studies (QUIPS) tool (Supplementary Table 2, available on the *Arthritis Care & Research* website at http://onlinelibrary.wiley.com/doi/10.1002/acr.24587). Disagreements were again discussed and resolved by a third reviewer when appropriate. Quality assessment was not used as a basis for excluding studies.

### Summary measures

The reporting of outcomes was expected to be highly heterogeneous; thus, a number of summary measures were recorded depending on the outcome of interest. For all‐cause mortality, hazard ratios (HRs), odds ratios (ORs), incidence rates, and survival percentages were deemed acceptable. For our health‐related outcomes of interest, we expected these to be measured using validated quantitative questionnaires, such as the health assessment questionnaire (HAQ), Short Form 36 (SF‐36), and EuroQol 5‐domain (EQ‐5D) questionnaire, with analyses involving various types of regression models. We therefore accepted regression model outputs, such as β coefficients and ORs.

### Synthesis of results

Studies included in the review were not considered sufficiently similar to perform a meta‐analysis. This was due to between‐study variations in the measures of multimorbidity used, whether such measures were treated as continuous or categorical variables, statistical analyses performed, and the adjusted variables (Tables 1 and 2). A narrative synthesis of findings from included studies was therefore conducted.

**Table 1:** Summary of synthesis methods and results for included studies assessing all‐cause mortality*

Author, year (ref.)	Outcome(s) measured	Multimorbidity measure (continuous/categorical)	Synthesis method	Standardized outcome metric	Synthesis findings (as reported)	Significant association (Yes/No, *P*)	Summary of findings
De Vera et al, 2012 (13)	All‐cause mortality	CCI (continuous: mean ± SD 1.0 ± 1.3)	Cox proportional hazards regression	HR (95% CI)	Unadjusted HR: 1.36 (1.29–1.43) Adjusted HR: 1.18 (1.10–1.25)	Yes, *P* < 0.0001 (unadjusted and adjusted).	Higher CCI score is a significant predictor of increased mortality risk.
England et al, 2016 (14)	All‐cause mortality	RDCI score (continuous: mean ± SD: 2.4 ± 1.7)	Cox proportional hazards regression	HR (95% CI)	Age‐adjusted HR: 1.15 (1.12–1.19) Multivariable HR: 1.14 (1.09–1.20)	Yes, *P* < 0.01 (age‐adjusted and multivariable).	RDCI score is associated with an increased risk for all‐cause mortality.
Fatima et al, 2020 (15)	All‐cause mortality	RDCI score (continuous: mean ± SD: 1.2 ± 1.3)	Discrete‐time proportional hazards models	Hazard OR (95% CI)	Unadjusted hazard OR: 1.60 (1.36–1.87)	Yes, *P* not stated.	All‐cause mortality was independently associated with more comorbidities.
Mikuls et al, 2011 (16)	All‐cause mortality	Condition count (continuous: mean ± SD: 2.1 ± 1.4)	Cox proportional hazards regression	HR (95% CI)	Age‐adjusted HR: 1.23 (0.98–1.54) Multivariate HR: 1.18 (0.99–1.41)	No, *P* not stated.	Comorbidity was not independently associated with a higher mortality risk in men with RA.
Navarro‐Cano et al, 2003 (17)	All‐cause mortality	CCI (categorical: 1, 2, >2) COMDUSOI (categorical: <40, 40–60, >60)	Kaplan‐Meier survival curves	Log‐rank *X* ^2^ (with 2 degrees of freedom)	CCI: 33.18 COMDUSOI: 29.24	Yes, *P* ≤ 0.0001 (CCI and COMDUSOI).	Higher comorbidity score was associated with a lower probability of survival.
Nikiphorou et al, 2020 (18)	All‐cause mortality	CCI (continuous: mean ± SD: 1.68 ± 1.06), RDCI (continuous: mean ± SD: 1.63 ± 1.48)	Cox proportional hazards regression	HR (95% CI)	Unadjusted: CCI 1.53 (1.21–1.93) RDCI 1.50 (1.20–1.87) Adjusted: CCI 1.30 (0.99–1.70) RDCI 1.26 (1.00–1.59)	Yes, unadjusted: CCI and RDCI *P* < 0.001. Yes, adjusted: RDCI *P* = 0.049. No, Adjusted: CCI *P* = 0.057.	Higher CCI and RDCI scores were both significantly associated with an increased risk of all‐ cause mortality in unadjusted analyses. This was also the case for RDCI in adjusted analysis.
Pedersen et al, 2018 (19)	All‐cause mortality	Condition count (categorical: 0, 1, 2, 3)	Cox proportional hazards regression	HR (95% CI)	Univariate HR: CCI 0 = 1, 1 = 2.34 (1.68–3.25), 2 = 4.61 (2.55–8.33), 3 = 12.22 (3.82–39.07) Multivariate HR: CCI 0 = 1, 1 = 1.64 (1.17–2.29), 2 = 3.32 (1.80–6.12), 3 = 5.48 (1.67‐17.94)	Yes, *P* = 0.000 (univariate) and *P* = 0.004, 0.000, 0.005 (multivariate).	Number of comorbid conditions was significantly associated with mortality.
Sokka et al, 2004 (20)	All‐cause mortality	Condition count (continuous: mean ± SE: 1.9 ± 0.1)	Cox proportional hazards regression	HR (95% CI)	Multivariate HR: 1.23 (1.05–1.44)	Yes, *P* = 0.01.	Number of comorbidities is an independent predictor of mortality.
Yoshida et al, 2019 (21)	All‐cause mortality	MWI (continuous: mean ± SD: 4.2 ± 4.4)	Inverse probability weighting	HR (95% CI)	Adjusted HR: 1.25 (1.13–1.40)	Yes, *P* not stated.	MWI substantially accounted for the excess total mortality in women with RA.
Norton et al, 2013 (31)	All‐cause mortality	Condition count (NCom; categorical: none, 1, >1), CCI (categorical: 0, 1, >1), Age‐adjusted CCI (categorical: 0, 1, >1)	Cox proportional hazards regression	HR (95% CI)	Crude HR: Age‐adjusted CCI 1.78 (1.69–1.89), CCI 1.63 (1.44–1.85), NCom 1.24 (1.16–1.33) Adjusted HR: Age‐adjusted CCI 1.23 (1.09–1.39), CCI 1.29 (1.13–1.48), NCom 1.09 (1.02–1.17)	Yes, *P* not stated.	Comorbidity was significantly associated with risk of all‐cause mortality.

* 95% CI = 95% confidence interval; CCI = Charlson comorbidity index; COMDUSOI = comorbidity Duke Severity of Illness score due to comorbidities alone; HR = hazard ratio; MWI = multimorbidity weighted index; NCom = total number of all comorbidities; OR = odds ratio; RA = rheumatoid arthritis; RDCI = Rheumatic Disease Comorbidity Index.

**Table 2:** Summary of synthesis methods and results for included studies assessing functional status and/or quality of life*

Author, year (ref.)	Outcomes measured (as reported)	Multimorbidity measure (continuous/categorical)	Synthesis method	Standardized outcome metric	Synthesis findings (as reported)	Significant association (Yes/No, *P*)	Summary of findings
Hitchon et al, 2016 (22)	Functional status (HAQ)	Condition count (continuous: median 2 [IQR 2]), CCI (modified) (categorical: 0, 1, 2–3, >3), SACQ (modified) (continuous: median 2 [IQR 3])	Multivariate linear regression	β (95% CI)	Number of comorbid conditions: 0.06 (0.05–0.08), CCI (modified): 0.06 (0.03–0.10), SACQ (modified): 0.07 (0.05–0.12)	Yes, *P* < 0.0001. (Number of comorbid conditions, CCI and SACQ).	Comorbidity independently influences functional status (HAQ) at 1 year.
Van den Hoek et al, 2013 (23)	Physical functioning (HAQ; validated Dutch version, SF‐36 PCS)	Condition count (categorical: none, somatic, depression, somatic and depression)	Linear, mixed‐effects, random intercept model	Annual change (95% CI)	Annual change: Somatic comorbidity and comorbid depression: HAQ 0.018 (0.002, 0.033) SF‐36 PCS −0.369 (−0.650, −0.083)	Yes, *P* < 0.02 (HAQ) and *P* < 0.01 (SF‐36 PCS).	Difference in physical functioning between those with both somatic comorbidity and comorbid depression and patients without comorbidity increased between baseline and 11‐year follow‐up.
Kapetanovic et al, 2015 (24)	Disability (HAQ; validated Swedish version); functional impairment (SOFI)	CCI (continuous: mean ± SD in years 0, 5, 10, 15, and 20, respectively: 0.4 ± 0.9, 0.7 ± 1.3, 1.2 ± 2, 1.9 ± 2.3, and 2.2 ± 2.4)	Hierarchical regression models	β (regression coefficient [slope]) ± SE (R^2^ change [of coefficient of variation])	HAQ: AUC 0.06 ± 0.03 (0.061) SOFI: AUC 0.48 ± 0.25 (0.038)	Yes, *P* < 0.001 (HAQ) and *P* < 0.01 (SOFI).	Contribution of comorbidity over the entire follow‐up time (AUC) was minor. 0.5–6% for SOFI and 0.6–8% for HAQ
Michaud et al, 2011 (25)	Functional status (HAQ)	Computed comorbidity score (categorical: 0, 1, 2, 3, 4 or more)	Multivariable regression models	Difference (95% CI) in annual rate of HAQ increase or decrease	Comorbid conditions: 2: 0.010 (0.006–0.015), 3: 0.014 (0.008–0.021), 4 or more: 0.012 (0.005–0.020)	Yes, *P* < 0.05.	HAQ progression was independently associated with the number of comorbid conditions at baseline.
Nakajima et al, 2015 (26)	Disability (J‐HAQ); Quality of life (EQ‐5D)	Age‐adjusted CCI (categorical: 0, 1–2, 3–4, and ≥5)	Linear regression models	Adjusted difference (SE)	J‐HAQ at 1 year: Adjusted CCI: 0 = Reference, 1–2 = 0.32 (0.09), 3–4 = 0.45 (0.10), ≥5 = 0.45 (0.15) EQ‐5D at 1 year: Adjusted CCI: 0 = Reference, 1–2 = −0.081 (0.027), 3‐4 = −0.086 (0.030), ≥5 = −0.146 (0.043)	Yes, J‐HAQ: *P* < 0.0001, *P* < 0.0001, *P* = 0.003. EQ‐5D: *P* = 0.002, *P* = 0.004, *P* < 0.001.	Physical function and quality of life was significantly affected by presence of comorbidities at 1 year.
Pan et al, 2019 (27)	Functional disability (HAQ)	Condition count (categorical: none, 1, 2, or ≥3 comorbidities)	Multinomial logistic regression	Relative risk ratios (95% CI)	Presence of 2 comorbidities versus no comorbidity: High–moderate (HAQ trajectory group): 2.49 (1.16–5.34), Severe: 4.76 (2.23–10.13), Very severe: 3.29 (1.34–8.04) Presence of ≥3 comorbidities versus no comorbidity: High–moderate (HAQ trajectory group): 11.98 (1.53–94.01), Severe: 24.16 (3.12–187.1), Very severe: 21.58 (2.58–180.2)	Yes, *P* not stated.	More comorbidity was associated with higher HAQ trajectory group.
Radner et al, 2010 (28)	Physical function (HAQ)	Age‐adjusted CCI (categorical: 0, 1–2, 3–4, ≥5)	GLM; EMM	GLM model R^2^; EMM of time‐averaged HAQ	GLM: R^2^ = 0.48; EMM: CCI 0 = 0.84, CCI 1–2 = 0.88, CCI 3–4 = 1.14, CCI ≥5 = 1.48	Yes, *P* < 0.001 (GLM) and *P* < 0.001 (EMM).	Physical disability worsens with increasing levels of comorbidity.
Radner et al, 2011 (29)	Functional disability (HAQ); Quality of life (SF‐36)	Age‐adjusted CCI (categorical: 0, 1–2, 3–4, 5–9)	GLM	GLM model R^2^	HAQ (GLM: R^2^ = 0.48) SF‐36 (GLM: R^2^ = 0.36)	HAQ: Yes, *P* < 0.03 (ANOVA); *P* < 0.001 (GLM). SF‐36: Yes, *P* < 0.001 (GLM).	HAQ and physical domains of SF‐36 equally affected by comorbidities.
Rupp et al, 2006 (30)	Disability (VDF); Health‐related quality of life (RAND‐36)	Condition count (continuous: somatic comorbidity, mean ± SD 1.1 ± 1.3 and range 0–7; and psychological comorbidity, mean ± SD 12.3 ± 9.2 and range 0–49	Multivariate logistic regression	OR (95% CI)	Disability: Somatic comorbidity = 1.2 (1.0–1.5), Psychological comorbidity = 1.1 (1.1–1.1) HRQoL PCS: Somatic comorbidity = 1.4 (1.1–1.6), Psychological comorbidity = 1.1 (1.0–1.1) HRQoL MCS: Somatic comorbidity = 1.1 (0.9–1.4), Psychological comorbidity = 1.3 (1.2–1.4)	Disability: Yes, *P* < 0.05 (somatic) and *P* ≤ 0.001 (psychological). HRQoL PCS: Yes, *P* ≤ 0.001 (somatic and psychological). MCS: Yes, *P* ≤ 0.001 (psychological). Somatic comorbidity not significant.	Somatic comorbidity appeared to be a risk factor for disability and PCS but not MCS. Psychological comorbidity increased risk for poor outcomes relating to disability, PCS and MCS.
Norton et al, 2013 (31)	Function (HAQ)	Age‐adjusted CCI (categorical: 0, 1, >1), CCI (categorical: 0, 1, >1), Condition count (NCom; categorical: none, 1, >1)	Piecewise mixed‐effects regression	β (95% CI)	Difference in change between baseline and 1 year: Age‐adjusted CCI −0.036 (−0.059, −0.013), CCI 0.005 (−0.057, 0.066), NCom 0.022 (−0.006, 0.051) Difference in rate of change between 1 and 10 years: Age‐adjusted CCI 0.016 (0.010, 0.022), CCI 0.022 (0.006, 0.038), NCom 0.011 (0.004, 0.019)	No, *P* not stated. (Difference in change between baseline and 1 year). Yes, *P* not stated. (Difference in rate of change between 1 and 10 years).	CCI, Age‐adjusted CCI, and NCom were all associated with increased rates of HAQ progression between 1 and 10 years follow‐up. Comorbidity was associated with increased rates of functional decline over 10 years.

* 95% CI = 95% confidence interval; ANOVA = analysis of variance; AUC = area under the curve; CCI = Charlson comorbidity index; EMM = estimated marginal means; EQ‐5D = EuroQol 5‐domain; GLM = generalized linear model; HAQ = health assessment questionnaire; HRQoL = health‐related quality of life; IQR = interquartile range; J‐HAQ = Japanese version of the HAQ; MCS = mental component score; NCom = total number of comorbidities; OR = odds ratio; PCS = physical component score; RAND‐36 = RAND 36‐Item Health Survey; SACQ = Self‐Administered Comorbidity Questionnaire; SF‐36 = Short Form 36; SOFI = signals of functional impairment; VDF = validated Dutch capacities of daily life questionnaire.

## RESULTS

### Study selection

**Figure 1:**
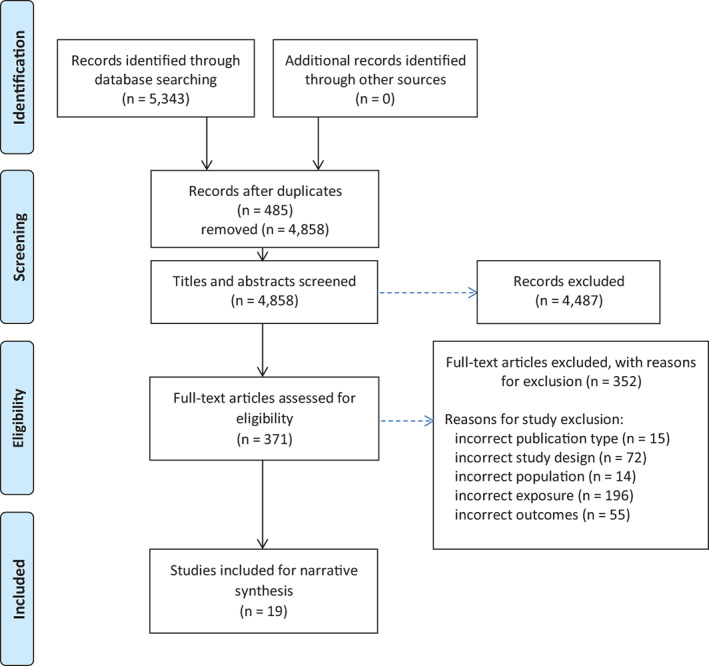
Flow diagram of the included studies according to the 2009 Preferred Reporting Items for Systematic Reviews and Meta‐Analyses protocol.

The total number of articles identified using our search strategy was 5,343. Of these studies, 19 met eligibility criteria and were included in the present review. The full study selection process, including reasons for exclusion at each screening stage, is shown in the 2009 PRISMA flow diagram (Figure 1) (11).

### Study characteristics

The 19 studies included in the present review are summarized in Table 3. Of these, 9 studies had all‐cause mortality as an outcome (13, 14, 15, 16, 17, 18, 19, 20, 21), and 9 showed functional status (22, 23, 24, 25, 26, 27, 28, 29, 30) with 3 of these studies also reporting quality of life (26, 29, 30). One study reported both all‐cause mortality and functional status (31). All studies were longitudinal observational studies conducted in high‐income countries, and the number of participants ranged from 183 to 18,485. All studies were conducted between 1985 and 2018, with follow‐up periods ranging from 6 months to 23 years.

**Table 3:** Summary of included studies*

Reference	Outcomes measured	Location	Study setting	Number of participants	Study period; follow‐up	Morbidity reporting method or source	Multimorbidity measure used
De Vera et al, 2012 (13)	All‐cause mortality	Canada	Population‐based RA cohort	4,102	1996–2006; 10 years	Medical records	CCI
England et al, 2016 (14)	All‐cause mortality	US	Veterans RA cohort	1,652	2003–2013; 10 years	Medical records	RDCI
Fatima et al, 2020 (15)	All‐cause mortality	Canada	RA inception cohort	1,724	2007–2017; 10 years	Self‐report	RDCI
Mikuls et al, 2011 (16)	All‐cause mortality	US	Veterans RA cohort	1,015	2002–2009; 7 years	Medical records	Condition count
Navarro‐Cano et al, 2003 (17)	All‐cause mortality	US	Outpatient rheumatology clinic	779	1996–2002; 6 years	Medical records; Self‐report	CCI; COMDUSOI
Nikiphorou et al, 2020 (18)	All‐cause mortality	UK	Primary care‐based population	6,591	Date of diagnosis–2017; 3 years	Medical records	CCI; RDCI
Pedersen et al, 2018 (19)	All‐cause mortality	Denmark	Rheumatology hospital	509	1995–2013; 18 years	Medical records	Condition count
Sokka et al, 2004 (20)	All‐cause mortality	Finland	Outpatient rheumatology clinic	1,095	2000–2002; 2 years	Self‐report	Condition count
Yoshida et al, 2019 (21)	All‐cause mortality	US	Nurses prospective cohort	1,007	Date of diagnosis–2018; 20 years (median)	Self‐report	MWI
Hitchon et al, 2016 (22)	Functional status	Canada	RA inception cohort	2,090	2006–2014; 1 year	Self‐report	CCI; condition count; SACQ
van den Hoek et al, 2013 (23)	Functional status	The Netherlands	Outpatient rheumatology clinic	882	1997–2008; 11 years	Self‐report	Condition count
Kapetanovic et al, 2015 (24)	Functional status	Sweden	Community‐based cohort	183	1985–2005; 20 years	Medical records; Self‐report	CCI
Michaud et al, 2011 (25)	Functional status	US	Community‐based cohort	18,485	1998–2009; 0.5–11 years	Self‐report	Condition count
Nakajima et al, 2015 (26)	Functional status; Quality of life	Japan	Outpatient rheumatology clinic	267	2010–2011; 0.5–1 year	Self‐report	Age‐adjusted CCI
Pan et al, 2019 (27)	Functional status	UK	Observational RA cohort	1,274	2002–2009; 3 years	Medical records; Self‐report	Condition count
Radner et al, 2010 (28)	Functional status	Austria	Outpatient rheumatology clinic	380	2007–2008; 1 year	Medical records	CCI; Age‐adjusted CCI; condition count
Radner et al, 2011 (29)	Functional status; Quality of life	Austria	Outpatient rheumatology clinic	380	2007–2008; 1 year	Medical records	Age‐adjusted CCI
Rupp et al, 2006 (30)	Functional status; Quality of life	The Netherlands	Outpatient rheumatology clinic	882	1997–2002; 5 years	Self‐report	Condition count
Norton et al, 2013 (31)	All‐cause mortality; Functional status	UK	Community‐based cohort	1,460	1986–2009; 23 years	Medical records; Self‐report	CCI; Age‐adjusted CCI; condition count

* CCI = Charlson comorbidity index; COMDUSOI = Duke Severity of Illness score due to comorbidities alone; MWI = multimorbidity weighted index; RA = rheumatoid arthritis; RDCI = Rheumatic Disease Comorbidity Index; SACQ = Self‐Administered Comorbidity Questionnaire.

### Population

Key participant demographic characteristics from each included study are summarized in Table 4. Of the 19 included studies, 16 studies reported the age of participants as a mean, ranging from 52 to 66.6 years old (13, 14, 15, 16, 18, 21, 22, 23, 25, 27, 28, 29, 30, 31), with 2 studies reporting median ages of 63 and 63.7 years (19, 27). The age of participants in the remaining study could not be accurately determined (17). Two studies restricted their analyses to male participants only (14, 16), with 1 study restricting theirs to female participants only (21). The percentage of female participants ranged from 60% to 88% across the remaining 16 studies (13, 15, 17, 18, 19, 20, 22, 23, 24, 25, 26, 27, 28, 29, 30, 31). Only 6 of the 19 included studies reported on ethnicity (15, 16, 17, 18, 21, 22), with the percentage of White participants in these studies ranging from 34.9% to 97.4%. Socioeconomic status (SES) was only reported in 2 of 19 studies (23, 30), with most participants (~60.1%) having a medium SES in both studies.

**Table 4:** Summary of key participant demographic characteristics*

Author, year (ref.)	Age†	Sex (%)	Ethnicity/race (as reported), no. (%)	Socioeconomic status (as reported), no. (%)
De Vera et al, 2012 (13)	66.6 ± 10.4	Female (60); male (40)	Not reported	Not reported
England et al, 2016 (14)	64.6 ± 10.4	Female (0); male (100)	Not reported	Not reported
Fatima et al, 2020 (15)	55.0 ± 15.0	Female (72); male (28)	White or European = 1,467 (85.0); Aboriginal = 74 (4.0)	Not reported
Mikuls et al, 2011 (16)	65.0 ± 11.0	Female (0); male (100)	White race (79.0)	Not reported
Navarro‐Cano et al, 2003 (17)	Unclear	Female (70.6); male (29.4)	Hispanic = 434 (55.7); White = 272 (34.9); African American = 53 (6.8); Asian = 14 (1.8); Other ethnic group = 6 (0.8)	Not reported
Nikiphorou et al, 2020 (18)	59.8 ± 15.8	Female (67.5); male (32)	White = 4,883 (91.6); Asian = 255 (4.8); Black = 128 (2.4); Mixed ethnicity = 26 (0.5); Other = 36 (0.7)	Not reported
Pedersen et al, 2018 (19)	Median 63.0 (IQR 53.0–71.0)	Female (68); male (32.4)	Not reported	Not reported
Sokka et al, 2004 (20)	62.4 ± SE 0.4	Female (71); male (29)	Not reported	Not reported
Yoshida et al, 2019 (21)	60.3 ± 10.3	Female (100); male (0)	White = 981 (97.4)	Not reported
Hitchon et al, 2016 (22)	53.5 ± 15.25	Female (73); male (27)	White = 1,691 (81.0)	Not reported
van den Hoek et al, 2013 (23)	59.3 ± 14.8	Female (71.9); male (28.1)	Not reported	Low = 220 (24.9); Medium = 526 (59.6); High = 123 (13.9); Missing = 13 (1.5)
Kapetanovic et al, 2015 (24)	52 ± 12	Female (63); male (37)	Not reported	Not reported
Michaud et al, 2011 (25)	60.0 (not reported)	Female (76.6); male (23.3)	Not reported	Not reported
Nakajima et al, 2015 (26)	Median 63.7 (IQR 55.7–70.4)	Female (88); male (12)	Not reported	Not reported
Pan et al, 2019 (27)	61.1 ± 12.3	Female (71.4); male (28.6)	Not reported	Not reported
Radner et al, 2010 (28)	60.7 ± 13.2	Female (80.5); male (19.5)	Not reported	Not reported
Radner et al, 2011 (29)	60.7 ± 13.2	Female (80.5); male (19.5)	Not reported	Not reported
Rupp et al, 2006 (30)	59.8 ± 14.8	Female (71.9); male (28.1)	Not reported	High = 123 (14.2); Medium = 526 (60.5); Low = 220 (25.3)
Norton et al, 2013 (31)	55.3 ± 14.6	Female (66.4); male (33.6)	Not reported	Not reported

* IQR = interquartile range.

† Unless indicated otherwise, values shown are the mean ± SD years.

### Exposure

Comorbid LTCs were recorded at baseline in 11 of the 19 included studies (13, 14, 16, 18, 19, 22, 25, 26, 28, 29, 31) and summarized in Supplementary Table 3, available on the *Arthritis Care & Research* website at http://onlinelibrary.wiley.com/doi/10.1002/acr.24587. Where reported, the most common comorbidities across studies were hypertension, diabetes mellitus, chronic pulmonary disease(s), and depression. The measurement of multimorbidity varied across the included studies, as summarized in Table 3. Seven of the 19 studies used a simple count of LTCs (with the maximum number considered ranging from 8 to 23 LTCs) as reported by the study participants and/or verified by medical records (16, 19, 20, 23, 25, 27, 30). The Charlson comorbidity index (32) was used alone in 4 of 19 studies (13, 24, 26, 29), again using self‐reported LTCs and/or medical record verification. The Rheumatic Disease Comorbidity Index (RDCI) was used alone in 2 other studies (14, 15), and another study used the multimorbidity weighted index (MWI) (21). The remaining 5 studies used a combination of measures including the Duke Severity of Illness score due to comorbidities alone with Charlson comorbidity index (17), Charlson comorbidity index and RDCI (18), Self‐Administered Comorbidity Questionnaire, Charlson comorbidity index and LTC count (22), and Charlson comorbidity index with LTC count (28, 31). This variation in how multimorbidity was measured and reported across studies further highlights the heterogeneous nature of the included studies.

### Findings from included studies

#### All‐cause mortality

Despite multimorbidity measures and statistical analyses varying between studies, multimorbidity was found to be a significant predictor of all‐cause mortality in people with RA in 9 of 10 included studies that had all‐cause mortality as an outcome. Methods and results from each study are summarized in Table 1. Cox proportional hazards regression model was used in 7 studies with the HR representing the independent risk of all‐cause mortality associated with multimorbidity. Of these 7 studies, 6 demonstrated a significant association between multimorbidity and higher risk of all‐cause mortality in RA populations (13, 14, 18, 19, 20, 31), whereas 1 study showed no significant association (16). Another study used inverse probability weighting as an alternative to the standard Cox model to estimate the effect of post‐diagnosis MWI on all‐cause mortality in women with RA, finding MWI substantially accounted for excess mortality (21). Additionally, in 2020, Fatima et al used discrete‐time proportional hazards models with the resulting significant hazard OR indicating that an increased number of comorbidities was independently associated with all‐cause mortality (15). Finally, Navarro‐Cano and colleagues used the Kaplan‐Meier method to estimate survival differences between participants grouped according to Charlson comorbidity index score, with an increase in Charlson comorbidity index score significantly lowering the probability of survival (17).

#### Other health‐related outcomes

Ten of 19 studies included in the present review explored the association between multimorbidity and the health‐related outcomes of interest (functional status and quality of life) in people with RA (22, 23, 24, 25, 26, 27, 28, 29, 30, 31). All 10 studies reported significant associations between multimorbidity and reduced functional status, with 3 of these also exploring and reporting a further association with impaired quality of life (26, 29, 30).

#### Functional status

Functional status was measured by HAQ in 9 of 10 studies, including validated Dutch (23), Swedish (24), and Japanese (26) versions that encompass culturally appropriate modifications. The physical component summary score of the SF‐36 was also used in one study along with the HAQ (23). Kapetanovic et al further developed and included a performance‐based instrument in their study together with the HAQ called Signals of Functional Impairment (SOFI) to assess function of the hands, upper extremities, and lower extremities (24). Another study used the validated Dutch capacities of daily life questionnaire (VDF) to measure disease impact in terms of disability (30). Statistical analyses were highly heterogeneous across studies and included multiple linear, logistic, and mixed‐effects regression models (methods and results summarized in Table 2).

Multimorbidity was found to significantly influence functional status when measured by the HAQ and independently contributed to functional decline over time in all 10 studies (22, 23, 24, 25, 26, 27, 28, 29, 30, 31). One study reported somatic comorbidity (covering groups of LTCs—specifically, lung diseases, CVD, diabetes mellitus, gastrointestinal diseases, cancer, kidney diseases, chronic infections, gallbladder and liver diseases, chronic back complaints, skin diseases, thyroid gland diseases, and neurologic diseases) and psychological comorbidity (depressive symptoms specifically) to be risk factors for increased disability (30). In people with RA, somatic comorbidity and depression were especially detrimental to physical functioning over time when compared to individuals with RA alone, as measured by the validated Dutch version of HAQ (23). Multimorbidity was also shown to significantly contribute to functional impairment (measured by SOFI) over 20 years, although this contribution was minimal (0.5–6%) (24). The independent effect of different multimorbidity levels when measured by age‐adjusted Charlson comorbidity index on physical disability (measured by the HAQ) was reported in 3 studies, with increasing age‐adjusted Charlson comorbidity index levels significantly affecting physical function (26) and a consistent increase in physical disability seen with higher age‐adjusted Charlson comorbidity index levels (28, 29). Furthermore, in 2019, Pan and colleagues identified trajectory groups of disability scores (determined by the HAQ) over a 3‐year period in people with RA who had moderate disease activity. The trajectories of HAQ scores over follow‐up were divided into 7 groups and were related to HAQ at baseline. These groups were classified as “very‐low,” “low,” “low‐moderate,” “moderate,” “high‐moderate,” “severe,” and “very‐severe,” with increased comorbidity independently associated with the higher HAQ trajectory groups (high‐moderate, severe, and very‐severe) (27).

#### Quality of life

Quality of life was measured using 3 distinct instruments across 3 studies, namely the EQ‐5D questionnaire (26), SF‐36 (29), and a validated Dutch version of the RAND 36‐Item Health Survey (30). Using the EQ‐5D over a one‐year observation period, Nakajima et al demonstrated in 2015 that increasing age‐adjusted Charlson comorbidity index scores (1–2, 3–4, and ≥5) significantly lowered quality of life when compared to the reference score (age‐adjusted Charlson comorbidity index level of 0) (26). In another study, the mental component score (MCS) of the SF‐36, which aggregates the following domains: vitality, social function, emotional role, and mental health, was unaffected by age‐adjusted Charlson comorbidity index level. However, the physical component score (PCS), which aggregates the domains of physical function, physical role, bodily pain, and general health perception, decreased in a linear fashion within the 4 groups of age‐adjusted Charlson comorbidity index scores (0, 1–2, 3–4 and ≥5), indicating an association between multimorbidity and poorer physical‐related quality of life (29). The final study to assess quality of life computed an MCS using the Dutch version of the RAND 36‐Item Health Survey, finding that psychological comorbidity, specifically depressive symptoms, consistently increased the risk for poor outcomes and also hampered good outcomes with respect to the MCS. Interestingly, somatic comorbidity was not a risk factor with respect to the MCS and appeared to be associated with good outcomes on the MCS (30).

#### Quality appraisal of included studies

The quality of each study was assessed and is summarized in Supplementary Table 4, available on the *Arthritis Care & Research* website at http://onlinelibrary.wiley.com/doi/10.1002/acr.24587. Of the included studies, the rating of risk of bias across domains was generally low based on assessment using the QUIPS tool. The rating of reporting was adequate across most studies allowing for risk of bias to be determined. Included studies were therefore deemed to be of good quality.

## DISCUSSION

This systematic review is the first, to our knowledge, to evaluate the existing literature to determine the association between multimorbidity and multiple adverse health‐related outcomes in people with RA. Our findings show that multimorbidity is significantly associated with higher mortality, poorer functional status, and lower quality of life in people with RA across a range of locations, study settings, and sample sizes.

Of the included studies, only Mikuls et al found no association between multimorbidity and all‐cause mortality (16). The study focused on a population of male military service veterans with RA and stated that the findings were not generalizable to other populations, including women (veterans and non‐veterans) with RA and non‐veteran men with RA. The limitations of the study also highlight a lack of sensitivity in the capturing of mortality events; thus, the authors acknowledge that the mortality burden may be underestimated in this population.

Included studies were diverse in their methodologies, especially in how multimorbidity was measured. Numerous indices exist with no universally agreed measure to quantify multimorbidity. There is little guidance on choosing the most suitable measure to use when predicting risk of adverse health‐related outcomes due in part to insufficient knowledge and understanding of what optimal management is to those living with multimorbidity (33). Condition counts are deemed appropriate for use in the absence of validated measures or when multiple outcomes or populations are being considered (34). However, these are often based on prespecified lists of LTCs that can vary between studies; thus, some conditions may not be captured. Some lists may also include LTCs that carry different importance in terms of their strength of individual association with adverse outcomes. Careful consideration should be given to both the range of conditions to be included and the research objectives as some conditions may impact the studied cohort or outcomes of interest more than others (e.g., depression and quality of life) (35). Weighted measures may therefore be more useful for certain outcomes, particularly when assessing the added burdens associated with multimorbidity (36).

The types of morbidities investigated varied across the included studies, with many recording physical conditions only (Supplementary Table 3, available on the *Arthritis Care & Research* website at http://onlinelibrary.wiley.com/doi/10.1002/acr.24587). Indeed, there is an abundance of literature detailing the increased prevalence of CVD in people with RA as a result of shared inflammatory pathways and risk profiles, including smoking and physical inactivity (6). In contrast, Dougados et al reported depression to be the most commonly observed comorbidity in RA (36). Despite this, many of the included studies did not have mental health comorbidities in their lists of conditions, and mental health conditions were also not featured in Charlson comorbidity index scoring (32). Van den Hoek and colleagues’ study in 2013 was the only one included in the present review that investigated the contribution of physical and mental health comorbidities individually and then in combination, showing comorbid depression to be a consistent predictive factor across all health‐related outcomes in RA with and without the presence of physical comorbidity (23). This suggests that future studies should also focus on the impact of mental health conditions, especially as shared pathophysiologic mechanisms with RA have been suggested, and it is unlikely that any single biologic pathway would account for all links between multimorbidity and adverse health‐related outcomes (37).

In 2019, Yoshida et al observed a more rapid accumulation of morbidities (measured by the MWI) after incident RA diagnosis in a female‐only cohort when compared to matched non‐RA comparators drawn from the same population. This accumulating multimorbidity accounted for much of the excess mortality, with cardiovascular‐ and respiratory‐related deaths being more common in those with RA (21). While the generalizability of these findings to more diverse RA populations requires further evidence, the study by Yoshida and colleagues nevertheless highlights the adverse impact of multimorbidity on mortality in people with RA, while emphasizing the importance of early detection and sustained monitoring of multimorbidity in those with RA, preferably by a multidisciplinary team working in collaboration with patients and caregivers.

This systematic review was limited by the clinical and methodologic heterogeneity of the included studies, which meant that a meta‐analysis could not be performed. The heterogeneity statistic (I^2^) describes the percentage of variation across studies due to heterogeneity (clinical and methodologic) rather than random sampling error (38). However, it has been suggested that this preferred method of measuring between‐study consistency has a substantial bias, in addition to being imprecise, when the number of studies being assessed is small (39). Only 2 of 19 studies included in the present systematic review shared the same outcome (all‐cause mortality), multimorbidity measure (Charlson comorbidity index; continuous variable), statistical analysis (Cox proportional hazards regression), and standardized outcome metric (HR; 95% confidence interval) (13, 18). In view of this, it was felt that a meta‐analysis may give misleading results, and thus a narrative synthesis of findings was conducted instead. Studies included in the present review were also restricted to those published in the English language, which could be regarded as a limitation, although there is evidence to suggest that this is not detrimental to the quality of a systematic review (40).

Multimorbidity in people with RA was shown to be associated with higher mortality and poorer health‐related outcomes in the present systematic review. Our findings suggest that the variation in measuring multimorbidity for health research purposes appears to exclude or fail to fully capture the impact of many mental health conditions in those with RA and multimorbidity. Furthermore, the current understanding of what combinations of concurrent LTCs are associated with poorer outcomes in people with RA is insufficient. Studies examining quality of life as an outcome were lacking, as were those involving more diverse RA populations (e.g., younger participants, ethnic minorities, and those with lower socioeconomic status). Further research addressing such evidence gaps is essential in order to inform shared management models to ensure appropriate multidisciplinary care that is specific to individuals.

## AUTHOR CONTRIBUTIONS

All authors were involved in drafting the article or revising it critically for important intellectual content, and all authors approved the final version to be submitted for publication. Ms. Canning had full access to all data in the study and takes responsibility for the integrity of the data and the accuracy of the data analysis.

### Study conception and design

Canning, Siebert, Jani, Mair, Nicholl.

### Acquisition of data

Canning, Siebert, Jani, Harding‐Edgar, Kempe, Mair, Nicholl.

### Analysis and interpretation of data

Canning, Siebert, Jani, Mair, Nicholl.

## Supporting information


**Supplementary Table 1** Summary of inclusion and exclusion criteria used during the study selection process. LTC(s) = long‐term condition(s); RA = rheumatoid arthritis.
**Supplementary Table 2.** Quality in prognosis studies (QUIPS) tool used to assess the risk of bias in individual studies.
**Supplementary Table 3.** Comorbidities (%) reported at baseline for included studies. CCI = Charlson comorbidity index; CCI_A_ = age‐adjusted CCI; COMDUSOI = comorbidity duke severity of illness checklist; COPD = chronic obstructive pulmonary disease; CVD = cardiovascular disease; GI = gastrointestinal; MI = myocardial infarction; MWI = multimorbidity weighted index; OA = osteoarthritis; RDCI = rheumatic disease comorbidity index.
**Supplementary Table 4.** Summary of quality assessment using the Quality in prognosis studies (QUIPS) tool.
