# Supplementary material for: Examining the Relationship Between Rheumatoid Arthritis, Multimorbidity, and Adverse Health‐Related Outcomes: A Systematic Review
Source: Arthritis Care Res (Hoboken). 2022 May 27;74(9):1500–12. doi: 10.1002/acr.24587 (PMC11475560; doi:10.1002/acr.24587)
Supplement: Supplementary file 1 — Supplementary Table 1 Summary of inclusion and exclusion criteria used during the study selection process. LTC(s) = long‐term condition(s); RA = rheumatoid arthritis. Supplementary Table 2. Quality in prognosis studies (QUIPS) tool used to assess the risk of bias in individual studies. Supplementary Table 3. Comorbidities (%) reported at baseline for included studies. CCI = Charlson comorbidity index; CCIA = age‐adjusted CCI; COMDUSOI = comorbidity duke severity of illness checklist; COPD = chronic obstructive pulmonary disease; CVD = cardiovascular disease; GI = gastrointestinal; MI = myocardial infarction; MWI = multimorbidity weighted index; OA = osteoarthritis; RDCI = rheumatic disease comorbidity index. Supplementary Table 4. Summary of quality assessment using the Quality in prognosis studies (QUIPS) tool. [file ACR-74-1500-s001.docx]

<<tbt>>**Table 1.** <<tbn>>Summary of synthesis methods and results for included studies assessing all-cause mortality*

| <<tbch>>  Author, year (ref.) | Outcome(s) measured | Multimorbidity measure (continuous/categorical) | Synthesis method | Standardized outcome metric | Synthesis findings  (as reported) | Significant association (Yes/No, *P*) | Summary of findings |
| --- | --- | --- | --- | --- | --- | --- | --- |
| <<tb>> |  |  |  |  |  |  |  |
| De Vera et al, 2012 (13) | All-cause mortality | CCI (continuous: mean ±  SD 1.0 ± 1.3) | Cox proportional  hazards regression | Hazard ratio  (95% CI) | Unadjusted HR:  1.36 (1.29–1.43)  Adjusted HR:  1.18 (1.10–1.25) | Yes, *P* < 0.0001  (unadjusted  and adjusted). | Higher CCI score is a  significant predictor  of increased  mortality risk. |
| England et al, 2016 (14) | All-cause mortality | RDCI score (continuous:  mean ± SD: 2.4 ± 1.7) | Cox proportional  hazards regression | Hazard ratio  (95% CI) | Age-adjusted HR:  1.15 (1.12–1.19)  Multivariable HR:  1.14 (1.09–1.20) | Yes, *P* < 0.01  (age-adjusted and  multivariable). | RDCI score is  associated with an  increased risk for all-  cause mortality. |
| Fatima et al, 2020 (15) | All-cause mortality | RDCI score (continuous:  mean ± SD: 1.2 ± 1.3) | Discrete-time  proportional hazards  models | Hazard OR  (95% CI) | Unadjusted hazard OR:  1.60 (1.36–1.87) | Yes, *P* not stated. | All-cause mortality  was independently  associated with  more comorbidities. |
| Mikuls et al, 2011 (16) | All-cause mortality | Condition count  (continuous: mean ±  SD: 2.1 ± 1.4) | Cox proportional  hazards regression | Hazard ratio  (95% CI) | Age-adjusted HR:  1.23 (0.98–1.54)  Multivariate HR:  1.18 (0.99–1.41) | No, *P* < 0.1  (age-adjusted and  multivariate). | Comorbidity was not  independently  associated with a  higher mortality risk  in men with RA. |
| Navarro-Cano et al, 2003 (17) | All-cause mortality | CCI (categorical: 1, 2, >2)  COMDUSOI (categorical:  <40, 40–60, >60) | Kaplan-Meier  survival curves | Log-rank *X*^2^  (with 2 degrees  of freedom) | CCI: 33.18  COMDUSOI: 29.24 | Yes, *P* ≤ 0.0001 (CCI  and COMDUSOI). | Higher comorbidity  score was associated  with a lower  probability of  survival. |
| Nikiphorou et al, 2020 (18) | All-cause mortality | CCI (continuous: mean ±  SD: 1.68 ± 1.06),  RDCI (continuous: mean  ± SD: 1.63 ± 1.48) | Cox proportional  hazards regression | Hazard ratio  (95% CI) | Unadjusted:  CCI 1.53 (1.21–1.93)  RDCI 1.50 (1.20–1.87)  Adjusted:  CCI 1.30 (0.99–1.70)  RDCI 1.26 (1.00–1.59) | Yes, unadjusted: CCI  and RDCI *P* < 0.001.  Adjusted: RDCI  *P* = 0.049.  No, Adjusted: CCI  *P* = 0.057. | Higher CCI and RDCI  scored were both  significantly  associated with an  increased risk of all-  cause mortality in  unadjusted analyses.  This was also the  case for RDCI in  adjusted analysis. |
| Pedersen et al, 2018 (19) | All-cause mortality | Condition count  (categorical: 0, 1, 2, 3) | Cox proportional  hazards regression | Hazard ratio  (95% CI) | Univariate HR:  CCI 0 = 1, 1 = 2.34 (1.68–  3.25), 2 = 4.61 (2.55–8.33),  3 = 12.22 (3.82–39.07)  Multivariate HR:  CCI 0 = 1, 1 = 1.64 (1.17–  2.29), 2 = 3.32 (1.80–6.12),  3 = 5.48 (1.67-17.94) | Yes, *P* = 0.000 (univariate) and  *P* = 0.004, 0.000, 0.005 (multivariate). | Number of comorbid  conditions was  significantly  associated with  mortality. |
| Sokka et al, 2004 (20) | All-cause mortality | Condition count  (continuous: mean ± SE:  1.9 ± 0.1) | Cox proportional  hazards regression | Hazard ratio  (95% CI) | Multivariate HR:  1.23 (1.05–1.44) | Yes, *P* = 0.01. | Number of  comorbidities is an  independent  predictor of mortality. |
| Yoshida et al, 2019 (21) | All-cause mortality | MWI (continuous: mean ±  SD: 4.2 ± 4.4) | Inverse probability  weighting | Hazard ratio  (95% CI) | Adjusted HR:  1.25 (1.13–1.40) | Yes, *P* not stated. | MWI substantially  accounted for the  excess total mortality  in women with RA. |
| Norton et al, 2013 (31) | All-cause mortality | Condition count (NCom;  categorical: none, 1, >1),  CCI (categorical: 0, 1, >1),  Age-adjusted CCI  (categorical: 0, 1, >1) | Cox proportional  hazards regression | Hazard ratio  (95% CI) | Crude HR:  Adjusted CCI 1.78 (1.69–  1.89), CCI 1.63 (1.44–1.85),  NCom 1.24 (1.16–1.33)  Adjusted HR:  Adjusted CCI 1.23 (1.09–  1.39), CCI 1.29 (1.13–1.48),  NCom 1.09 (1.02–1.17) | Yes, *P* not stated. | Comorbidity was  significantly  associated with risk of  all-cause mortality. |

<<tbf>>

* 95% CI = 95% confidence interval; CCI = Charlson comorbidity index; COMDUSOI = comorbidity Duke Severity of Illness score due to comorbidities alone; HR = hazard ratio; MWI = multimorbidity weighted index; NCom = ●●; OR = odds ratio; RDCI = Rheumatic Disease Comorbidity Index.

<<tbt>>**Table 2.** <<tbn>> Summary of synthesis methods and results for included studies assessing functional status and/or quality of life*

| <<tbch>>  Author, year (ref.) | Outcomes measured  (as reported) | Multimorbidity measure (continuous/categorical) | Synthesis method | Standardized outcome metric | Synthesis findings  (as reported) | Significant association  (Yes/No, *P*) | Summary of findings |
| --- | --- | --- | --- | --- | --- | --- | --- |
| <<tb>> |  |  |  |  |  |  |  |
| Hitchon et al,  2016 (22) | Functional status (HAQ) | Condition count (continuous:  median 2 [IQR 2]), CCI  (modified) (categorical: 0, 1,  2–3, >3), SACQ (modified)  (continuous: median 2 [IQR 3]) | Multivariate linear regression | β (95% CI) | Number of comorbid  conditions: 0.06  (0.05–0.08), CCI  (modified): 0.06  (0.03–0.10), SACQ  (modified): 0.07  (0.05–0.12) | Yes, *P* < 0.0001.  (Number of comorbid  conditions, CCI and  SACQ). | Comorbidity independently  influences functional  status (HAQ) at 1 year. |
| van den Hoek et  al, 2013 (23) | Physical functioning  (HAQ; validated  Dutch version,  SF-36 PCS) | Condition count (categorical:  none, somatic, depression,  somatic and depression) | Linear, mixed-effects, random intercept model | Annual change (95% CI) | Annual change:  Somatic comorbidity  and comorbid  depression: HAQ  0.018 (0.002, 0.0033)  SF-36 PCS −0.369  (−0.650, −0.083) | Yes, *P* < 0.02 (HAQ)  and *P* < 0.01 (SF-36  PCS). | Difference in physical  functioning between  those with both somatic  comorbidity and  comorbid depression and  patients without  comorbidity increased  between baseline and  11-year follow-up. |
| Kapetanovic et al,  2015 (24) | Disability (HAQ;  validated Swedish  version); functional  impairment (SOFI) | CCI (continuous: mean ± SD in  years 0, 5, 10, 15, and 20,  respectively: 0.4 ± 0.9, 0.7 ±  1.3, 1.2 ± 2, 1.9 ± 2.3, and 2.2 ±  2.4) | Longitudinal regression models | β (regression coefficient [slope]) ± SE (R^2^ change [of coefficient of variation]) | HAQ: AUC 0.06 ± 0.03  (0.061)  SOFI: AUC 0.48 ± 0.25  (0.038) | Yes, *P* < 0.001 (HAQ)  and *P* < 0.01 (SOFI). | Contribution of  comorbidity over the  entire follow-up time  (AUC) was minor.  0.5–6% for SOFI and  0.6–8% for HAQ |
| Michaud et al,  2011 (25) | Functional status (HAQ) | Computed comorbidity score  (categorical: 0, 1, 2, 3, 4  or more) | Multivariable regression models | Difference (95% CI) in annual rate of HAQ increase or decrease | Comorbid conditions:  2: 0.010 (0.006–0.015),  3: 0.014 (0.008–0.021),  4 or more: 0.012 (0.005–  0.020) | Yes, *P* < 0.05. | HAQ progression was  independently associated  with the number of  comorbid conditions at  baseline. |
| Nakajima et al,  2015 (26) | Disability (J-HAQ); Quality of life  (EQ-5D) | Adjusted CCI (categorical: 0,  1–2, 3–4, and ≥5) | Linear regression models | Adjusted difference (SE) | J-HAQ at 1 year:  Adjusted CCI of 0 =  Reference, 1–2 = 0.32  (0.09), 3–4 = 0.45 (0.10),  ≥5 = 0.45 (0.15)  EQ-5D at 1 year:  Adjusted CCI of 0 =  Reference, 1–2 = −0.081  (0.027), 3-4 = −0.086  (0.030), ≥5 = −0.146  (0.043) | Yes,  J-HAQ: *P* < 0.0001,  *P* < 0.0001, *P* = 0.003.  EQ-5D: *P* = 0.002,  *P* = 0.004, *P* < 0.001. | Physical function and  quality of life was  significantly affected by  presence of comorbidities  at 1 year. |
| Pan et al, 2019  (27) | Functional disability (HAQ) | Condition count (categorical:  none, 1, 2, or ≥3 comorbidities) | Multinomial logistic regression | Relative risk ratios (95% CI) | Presence of 2 comorbidities  versus no comorbidity:  High–moderate  (HAQ trajectory group):  2.49 (1.16–5.34),  Severe: 4.76 (2.23–10.13),  Very severe: 3.29 (1.34–  8.04)  Presence of ≥3 comorbidities  versus no comorbidity:  High–moderate  (HAQ trajectory group):  11.98 (1.53–94.01),  Severe: 24.16 (3.12–187.1),  Very severe: 21.58 (2.58–  180.2) | Yes, *P* not stated. | More comorbidity was  associated with higher  HAQ trajectory group. |
| Radner et al, 2010  (28) | Physical function (HAQ) | Adjusted CCI (continuous: mean  ± SD 3.82 ± 1.6) | ANOVA; GLM | GLM model R^2^ | ANOVA: See Figure 1.  GLM: R^2^ = 0.48 | Yes, *P* < 0.01  (ANOVA), *P* < 0.001  (GLM). | Physical disability worsens  with increasing levels of  comorbidity. |
| Radner et al, 2011  (29) | Functional disability (HAQ); Quality of life (SF-36) | Adjusted CCI (categorical: 0, 1,  or 2, 3 or 4, ≥5) | ANOVA; GLM | Increase in HAQ value (time averaged) calculated by ANOVA; GLM model R^2^ | HAQ (ANOVA: See Figure  1; GLM: R^2^ = 0.48)  SF-36 (ANOVA: See Figure  1; GLM: R^2^ = 0.36) | HAQ: Yes, *P* < 0.03  (ANOVA); *P* < 0.001  (GLM).  SF-36: Yes, *P* < 0.05  (ANOVA); *P* < 0.001  (GLM). | HAQ and physical domains  of SF-36 equally affected  by comorbidities. |
| Rupp et al, 2006  (30) | Disability (VDF); Health-related quality of life (RAND-36) | Condition count (continuous:  somatic comorbidity, mean ±  SD 1.1 ± 1.3 and range 0–7;  and psychological comorbidity,  mean ± SD 12.3 ± 9.2 and  range 0–49 | Multivariate logistic regression | OR (95% CI) | Disability: Somatic  comorbidity = 1.2 (1.0–1.5),  Psychological comorbidity =  1.1 (1.1–1.1)  HRQoL PCS: Somatic  comorbidity = 1.4 (1.1–1.6),  Psychological comorbidity =  1.1 (1.0–1.1)  HRQoL MCS: Somatic  comorbidity = 1.1 (0.9–1.4),  Psychological comorbidity =  1.3 (1.2–1.4) | Disability: Yes, *P* < 0.05  (somatic) and *P* ≤ 0.001  (psychological).  HRQoL PCS: Yes,  *P* ≤ 0.001 (somatic and  psychological).  MCS: Yes, *P* ≤ 0.001  (psychological).  Somatic comorbidity  not significant. | Somatic comorbidity  appeared to be a risk  factor for disability and  PCS but not MCS.  Psychological comorbidity  increased risk for poor  outcomes relating to  disability, PCS and MCS. |
| Norton et al, 2013  (31) | Function (HAQ) | Adjusted CCI (categorical: 0,  1, >1), CCI (categorical: 0,  1, >1), Condition count  (NCom; categorical: none,  1, >1) | Piecewise mixed-effects regression | β (95% CI) | Difference in change between  baseline and 1 year:  Adjusted CCI −0.036 (−0.059,  −0.013), CCI 0.005 (−0.057,  0.066), NCom 0.022 (−0.006,  0.051)  Difference in rate of change  between 1 and 10 years:  Adjusted CCI 0.016 (0.010,  0.022), CCI 0.022 (0.006,  0.038), NCom 0.011 (0.004,  0.019) | No, *P* not stated.  (Difference in change  between baseline and  1 year).  Yes, *P* not stated.  (Difference in rate of  change between 1 and  10 years). | CCI, Adjusted CCI, and  NCom were all associated  with increased rates of  HAQ progression  between 1 and 10 years  follow-up.  Comorbidity was  associated with increased  rates of functional decline  over 10 years. |

<<tbf>>

* 95% CI = 95% confidence interval; ANOVA = analysis of variance; AUC = area under curve; CCI = Charlson comorbidity index; EQ-5D = EuroQol 5-domain; GLM = generalized linear model; HAQ = health assessment questionnaire; HRQoL = health-related quality of life; IQR = interquartile range; J-HAQ = ●●; MCS = mental component score; NCom = ●●; OR = odds ratio; PCS = physical component score; RAND-36 = RAND 26-Item Health Survey; SACQ = Self-Administered Comorbidity Questionnaire; SF-36 = Short Form 36; SOFI = signals of functional impairment; VDF = validated Dutch capacities of daily life questionnaire.

<<tbt>>**Table 3.** <<tbn>>Summary of included studies*

| <<tbch>>Reference | Outcomes measured | Location | Study setting | Number of participants | Study period;  follow-up | Morbidity reporting method or source | Multimorbidity measure used |
| --- | --- | --- | --- | --- | --- | --- | --- |
| <<tb>> |  |  |  |  |  |  |  |
| De Vera et al, 2021 (13) | All-cause mortality | Canada | Population-based RA cohort | 4,102 | 1996–2006;  10 years | Medical records | CCI |
| England et al, 2016 (14) | All-cause mortality | US | Veterans RA cohort | 1,652 | 2003–2013;  10 years | Medical records | RDCI |
| Fatima et al, 2020 (15) | All-cause mortality | Canada | RA inception cohort | 1,724 | 2007–2017;  10 years | Self-report | RDCI |
| Mikuls et al, 2011 (16) | All-cause mortality | US | Veterans RA cohort | 1,015 | 2002–2009;  7 years | Medical records | Condition count |
| Navarro-Cano et al, 2003 (17) | All-cause mortality | US | Outpatient rheumatology clinic | 779 | 1996–2002;  6 years | Medical records; Self-report |  |
| Nikiphorou et al, 2020 (18) | All-cause mortality | UK | Primary care-based population | 6,591 | Date of diagnosis–2017;  3 years | Medical records | CCI; COMDUSOI |
| Pedersen et al, 2018 (19) | All-cause mortality | Denmark | Rheumatology hospital | 509 | 1995–2013;  18 years | Medical records | Condition count |
| Sokka et al, 2004 (20) | All-cause mortality | Finland | Outpatient rheumatology clinic | 1,095 | 2000–2002;  2 years | Self-report | Condition count |
| Yoshida et al, 2019 (21) | All-cause mortality | US | Nurses prospective cohort | 1,007 | Date of diagnosis–2018;  20 years (median) | Self-report | MWI |
| Hitchon et al, 2016 (22) | Functional status | Canada | RA inception cohort | 2,090 | 2006–2014;  1 year | Self-report | CCI; condition count; SACQ |
| van den Hoek et al, 2013 (23) | Functional status | The Netherlands | Outpatient rheumatology clinic | 882 | 1997–2008;  11 years | Self-report | Condition count |
| Kapetanovic et al, 2015 (24) | Functional status | Sweden | Community-based cohort | 183 | 1985–2005;  20 years | Medical records; Self-report | CCI |
| Michaud et al, 2011 (25) | Functional status | US | Community-based cohort | 18,485 | 1998–2009;  0.5–11 years | Self-report | Condition count |
| Nakajima et al, 2015 (26) | Functional status; Quality of life | Japan | Outpatient rheumatology clinic | 267 | 2010–2011;  0.5–1 year | Self-report | Age-adjusted CCI |
| Pan et al, 2019 (27) | Functional status | UK | Observational RA cohort | 1,274 | 2002–2009;  3 years | Medical records; Self-report | Condition count |
| Radner et al, 2010 (28) | Functional status | Austria | Outpatient rheumatology clinic | 380 | 2007–2008;  1 year | Medical records | CCI; Age-adjusted CCI; condition count |
| Radner et al, 2011 (29) | Functional status; Quality of life | Austria | Outpatient rheumatology clinic | 380 | 2007–2008;  1 year | Medical records | Age-adjusted CCI |
| Rupp et al, 2006 (30) | Functional status; Quality of life | The Netherlands | Outpatient rheumatology clinic | 882 | 1997–2002;  5 years | Self-report | Condition count |
| Norton et al, 2013 (31) | All-cause mortality; Functional status | UK | Community-based cohort | 1,460 | 1986–2009;  23 years | Medical records; Self-report | CCI; Age-adjusted CCI; condition count |

<<tbf>>

* CCI = Charlson comorbidity index; COMDUSOI = Duke Severity of Illness score due to comorbidities alone; MWI = multimorbidity weighted index; RA = rheumatoid arthritis; RCDI = Rheumatic Disease Comorbidity Index; SACQ = Self-Administered Comorbidity Questionnaire.

<<tbt>>**Table 4.** <<tbn>>Summary of key participant demographic characteristics*

| <<tbch>>  Author, year (ref.) | Age† | Sex (%) | Ethnicity/race (as reported),  no. (%) | Socioeconomic status  (as reported), no. (%) |
| --- | --- | --- | --- | --- |
| <<tb>> |  |  |  |  |
| De Vera et al, 2012 (13) | 66.6 ± 10.4 | Female (60); male (40) | Not reported | Not reported |
| England et al, 2016 (14) | 60.7 ± 13.2 | Female (0); male (100) | Not reported | Not reported |
| Fatima et al, 2020 (15) | 55.0 ± 15.0 | Female (72); male (28) | White or European = 1,467 (85.0);  Aboriginal = 74 (4.0) | Not reported |
| Mikuls et al, 2011 (16) | 65.0 ± 11.0 | Female (0); male (100) | White race (79.0) | Not reported |
| Navarro-Cano et al, 2003 (17) | Unclear | Female (70.6); male (29.4) | Hispanic = 434 (55.7);  White = 272 (34.9);  African American = 53 (6.8);  Asian = 14 (1.8);  Other ethnic group = 6 (0.8) | Not reported |
| Nikiphorou et al, 2020 (18) | 59.8 ± 15.8 | Female (67.5); male (32.5) | White = 4,883 (91.6);  Asian = 255 (4.8);  Black = 128 (2.4);  Mixed race = 26 (0.5);  Other = 36 (0.7) | Not reported |
| Pedersen et al, 2018 (19) | Median 63.0  (IQR 53.0–71.0) | Female (67.6); male (32.4) | Not reported | Not reported |
| Sokka et al, 2004 (20) | Unclear | Female (71.6); male (28.4) | Not reported | Not reported |
| Yoshida et al, 2019 (21) | 60.3 ± 10.3 | Female (100); male (0) | White = 981 (97.4) | Not reported |
| Hitchon et al, 2016 (22) | 53.5 ± 15.25 | Female (73); male (27) | White = 1,691 (81.0) | Not reported |
| van den Hoek et al, 2013 (23) | 59.3 ± 14.8 | Female (71.9); male (28.1) | Not reported | Low = 220 (24.9);  Medium = 526 (59.6);  High = 123 (13.9);  Missing = 13 (1.5) |
| Kapetanovic et al, 2015 (24) | Unclear | Female (63.4); male (36.6) | Not reported | Not reported |
| Michaud et al, 2011 (25) | 60.0 (not reported) | Female (76.6); male (23.3) | Not reported | Not reported |
| Nakajima et al, 2015 (26) | Median 61.7  (IQR 52.3–69.2) | Female (84.2); male (15.8) | Not reported | Not reported |
| Pan et al, 2019 (27) | 61.1 ± 12.3 | Female (71.4); male (28.6) | Not reported | Not reported |
| Radner et al, 2010 (28) | 60.7 ± 13.2 | Female (80.5); male (19.5) | Not reported | Not reported |
| Radner et al, 2011 (29) | 60.7 ± 13.2 | Female (80.5); male (19.5) | Not reported | Not reported |
| Rupp et al, 2006 (30) | 59.8 ± 14.8 | Female (71.9); male (28.1) | Not reported | High = 123 (14.2);  Medium = 526 (60.5);  Low = 180 (20.6) |
| Norton et al, 2013 (31) | 55.3 ± 14.6 | Female (66.4); male (33.6) | Not reported | Not reported |

<<tbf>>

* IQR = interquartile range.

† Unless indicated otherwise, values shown are the mean ± SD years.
